# Associations of keratinocyte cancers with snp variants in the sonic hedgehog pathway

**DOI:** 10.1186/s12885-022-09565-6

**Published:** 2022-05-03

**Authors:** Astrid J. Rodriguez-Acevedo, Annika Antonsson, Upekha E. Liyanage, Maria Celia Hughes, Scott Gordon, Jolieke van der Pols, Adele C. Green

**Affiliations:** 1 The University of Queensland Diamantina Institute, The University of Queensland, Dermatology Research Centre, Brisbane, QLD Australia; 2 QIMR Berghofer Medical Research Institute, Royal Brisbane Hospital, Locked Bag 2000, Brisbane, QLD 4006 Australia; 3 Faculty of Medicine, The University of Queensland, Brisbane, Australia; 4 Faculty of Health, School of Exercise and Nutrition Sciences, Queensland University of Technology (QUT), Brisbane, Australia; 5 CRUK Manchester Institute and Faculty of Biology Medicine and Health, University of Manchester, Manchester Academic Health Science Centre, Manchester, UK

**Keywords:** BCC, SCC, Sonic Hedgehog pathway, Case–Control study, Genetics

## Abstract

**Background:**

Sonic Hedgehog (SHH) pathway dysregulation is implicated in basal cell carcinoma (BCC) development. To evaluate the possible wider role of SHH gene variants in skin carcinogenesis, we assessed associations of genes in the SHH pathway with lifetime development of any keratinocyte cancer (KC), and with developing either BCCs or squamous cell carcinomas (SCCs) exclusively, in a 25-year prospective, population-based study of 1,621 Australians.

**Methods:**

We genotyped 795 unrelated adults with available blood samples: 311 cases with any KC (186 developing BCCs-only, 55 SCCs-only, 70 BCCs and SCCs) and 484 controls. We compared allele frequencies of 158 independent SNPs across 43 SHH genes between cases and controls, and performed a gene-based analysis.

**Results:**

We found associations between SNP rs4848627 *(GLI2*) (related to DNA synthesis in keratinocytes) and development of any KC (OR = 1.53; 95% CI = 1.06–2.13, *P* < 0.01) and SCCs exclusively (OR = 2.12; 95%CI = 1.39–3.23, *P* < 0.01). SNP rs3217882 located in *CCND2* was associated with exclusive BCC development (OR = 1.43, CI = 1.12–1.82, *P* < 0.01). The gene-based analysis suggested an association of *PRKACG* (protein kinase cAMP-activated catalytic subunit gamma) with any KC (*P* = 0.013).

**Conclusion:**

We conclude that variants located in genes in the SHH pathway may are involved in SCC as well as BCC development.

**Supplementary Information:**

The online version contains supplementary material available at 10.1186/s12885-022-09565-6.

## Introduction

Keratinocyte cancers (KCs), comprising basal cell carcinomas (BCCs) and cutaneous squamous cell carcinomas (SCCs), are the most common cancers in fair-skinned people worldwide [1, 2]. The highest incidence rates of KCs are seen in white populations living in countries like New Zealand and Australia with high levels of solar ultraviolet (UV) radiation [3, 4]. Well-established risk factors associated with these skin cancers are light skin pigmentation, high sun exposure, and genetic factors [2, 5, 6]. Evidence from twin studies suggests the contribution from genetic inheritance is substantial [7].

In humans, the Sonic Hedgehog (SHH) signal transduction pathway is essential for embryonic development [8]. In skin, dysregulation of this signal transduction pathway is strongly implicated in the development of BCCs [9], and some laboratory evidence also suggests involvement with SCC development [10]. Specifically the SHH pathway is involved in cell cycle regulation, and an activated SHH pathway in adulthood results in stimulated cell growth and proliferation [11] associated consistently with sporadic BCCs [9]. Dysregulation of SHH-associated genes Patched 1 (*PTCH1*) and smoothened (*SMO*) has been observed in skin cancer and other malignant conditions [12]. Normally, the *PTCH1* gene serves as a negative regulator that controls the *SMO* gene by inhibition, which in turn binds to the glioma-associated oncogene (*GLI*), a transcription factor regulating apoptosis and angiogenesis [13]. However, details of the mechanisms by which the SHH pathway affects keratinocyte tumorigenesis remain to be elucidated. We therefore evaluated whether genes involved in the SHH pathway are associated with the development of BCCs exclusively, SCCs exclusively, and overall KC, in a 25-year prospective population-based study of skin cancer in Australian adults, using well-characterized phenotypes [14, 15].

## Materials and methods

### Study design

The Nambour Skin Cancer Study was a prospective cohort study of 2095 randomly selected residents of the Nambour township in subtropical Australia. The study was initiated in 1986 with skin examinations of all participants by dermatologists [16]. Skin cancers occurring 1987–1992 were collected prospectively by postal surveys and confirmed against medical records [17]. From 1992 to 1996, 1621 of the original 2095 participants took part in a randomized trial of daily sunscreen application and beta-carotene supplementation for skin cancer prevention [16, 18, 19]. All participants underwent an interview at trial baseline in which detailed dietary, medical, and occupational histories were recorded, and blood was collected at the end of the trial in 1996.

Dermatologists carried out full-body skin examinations of Nambour Study participants in 1992, 1994, 1996, and 2007. Clinically diagnosed skin cancers were confirmed histologically and skin cancers between clinical surveys were ascertained by linkage with the databases of local pathology laboratories [16, 20]. Therefore we had complete records of all histologically confirmed BCCs and SCCs of Nambour Skin Cancer Study participants from 1986–2007, and were able to distinguish people who in their lifetime to 2007 had exclusively developed BCCs, exclusively developed SCCs, or who had developed both types of KC, and controls who had never developed skin cancer [21]. This study was approved by the Human Research Ethics Committee of the QIMR Berghofer Medical Research Institute and all participants gave written informed consent.

### DNA extraction

DNA was extracted from blood samples by the Perkin Elmer Chemagic 360 extraction robot which uses automated magnetic bead technology. Sample quality (A_260/280_ ratio) and concentration were checked with a BioTek Synergy HT plate reader. A number of 69 samples were extracted previously using the QiAMP blood kit (Qiagen, Inc., Chadstone Centre, VIC, Australia) [15].

### Genotyping

Genotyping was carried out on 863 available blood samples using Illumina Global Screening Array (GSA) chips (model GSAMD-24v1-0–20,011,747; manifest revision A4) by the Human Genomics Facility (HuGeF) at Erasmus Medical Centre, Rotterdam. Genotypes were called using the Genotyping Module in Illumina GenomeStudio by standard procedures, with the following marker (SNP) exclusions: (1) AB R mean (Mean of the normalized R-values for the AB genotypes) < 0.2 (*N* = 0); (2) AB T mean < 0.2 or > 0.8, and clusters not distinguishable on manual inspection (*N* = 0); (3) GenTrain score (a measurement of quality of SNP calling) below 0.6 (*N* = 13,698). Further SNP quality control (QC) resulted in exclusion of further SNPs due to, (4) missingness > 5% (634); (5) Hardy Weinberg equilibrium (HWE) in PLINK 1.90, *p*-value < 10^–6^ (*N* = 20,699); (6) minor allele frequency (MAF) < 1% (*N* = 152,735); (7) heterozygous for > 1% of males (chromosome X, *N* = 493); (8) called for > 1% of females (chromosome Y, *N* = 276). 560 SNPs were also dropped due to duplication or failure to align uniquely, or at all, to the hg19 reference genome in the BLAST searches (URL https://www.well.ox.ac.uk/~wrayner/strand/). This resulted in retention of 500,655 out of the original 692,338 SNPs. Six samples whose genotype call rate was < 97% were dropped, leaving 857 participants after the QC stage. Of the 857 participants, 33 were siblings, and 29 were parent/child pairs. Of the related pairs, we deleted one participant from each pair. If one of the people in the pair had been affected by skin cancer, we retained that person. After removing the participants who were related, 795 remained for further analysis.

### SNPs associated with sonic hedgehog pathway

We used the KEGG pathways database [22, 23] to identify SNPs located in the Hedgehog signaling pathway. (Pathway identifier: hsa04340; Supplementary Table 5).

### SNPs association analysis

From the 500,655 SNPs resulting from genotyping, we extracted information for all SNPs located in 43 known Hedgehog signaling pathway genes. We selected only independent SNPs by using linkage disequilibrium (LD) pruning (using a 5 SNPs shifting window every 100 SNPs and a 0.2 LD threshold; we further excluded SNPs with minor allele frequency (MAF) < 5%).

We analysed three different histologically-confirmed case groups: (1) participants who had only BCCs (had never developed SCC); (2) those with a history of SCCs only (no ss); and (3) a combination of groups 1 and 2 plus those who with a history of both BCCs and SCCs (group 3 is henceforth referred to as “any KC” phenotype). Each of the three case groups was compared to the controls who had never developed BCCs or SCCs. We tested associations between extracted SNPs in the SHH pathway with BCCs, SCCs and KCs using a χ2 test to compare allele frequencies between cases and controls using PLINK v1.90 [24]. Bonferroni correction (0.05/158) was applied to account for the multiple comparisons. However, since this method might impose an unnecessary high penalty in association studies in particular those with a relatively small sample size like ours [25], we also calculated empirical *p*-values using a label-swapping permutation test implemented in PLINK with the –perm command [24]. Using the empirical *p*-values we considered a SNP associated with an outcome when *p* < 0.05.

### Gene-based analysis

Gene-based analysis was performed in MAGMA (Multi-marker analysis for genomic annotation) [26]. MAGMA uses SNP *p*-values to calculate a gene test-statistic as the mean of the χ2 statistics for all SNPs between the start and stop sites of a gene, correcting for gene size, number of SNPs in a gene, and LD [26]. We used the 1000 Genomes Build 37 reference data for gene annotation, and a 5 kb window to include neighbouring SNPs around each gene with potential regulatory roles in transcription.

### Sensitivity analysis

In the control group there were 56 participants whose skin cancer history prior to 1986 was uncertain (11 did not answer a question in the 1986 survey about whether they had a past skin cancer treated by a doctor; two persons were unsure whether they had a prior skin cancer; and 43 answered that they had had a skin cancer in the past but that this was not histologically confirmed). To evaluate potential misclassification of controls, we performed a sensitivity analysis by repeating the analyses without these 56 participants.

## Results

### Characteristics of the study population

We included 186 persons who developed BCCs exclusively, 55 who developed SCCs exclusively, and 70 persons who developed both BCCs and SCCs during the study period, as well as 484 controls who had not developed keratinocyte cancers. A total of 427 BCC lesions were diagnosed in the 186 participants with BCCs exclusively during the 1986–2007 study period. Of these, 22% (*n* = 92) were classified as aggressive BCC subtypes (namely: morphoeic, infiltrative, micronodular, basosquamous), 57% (*n* = 244) as non-aggressive subtypes (nodular, superficial), while for 21% (*n* = 91), subtypes were not available. All SCCs in study participants were localized invasive SCCs. Among the cases, 126 (41%) were aged ≥ 60 years, 147 (47%) were male, 162 (52%) had fair skin, and the majority of all study participants claimed predominantly European ancestry (99.5%) (Supplementary Table 1). The age distribution was significantly different between cases and controls (*p* < 0.05), with most cases (64%) aged 50 to 70 years and most controls (63%) aged 20 to 49 years. Our case group also included a higher number of individuals with fair skin colour (52% vs 40%) that were more likely to report that they “always burn” after exposure to acute sun (30% vs 15%), and more likely to have clinical elastosis of the neck (41% vs 26%) compared to controls.

### SNP association analysis

A total of 773 SNPs from the original extraction of the KEGG database were located across 43 genes in the SHH pathway. After excluding SNPs with MAF < 5% (*n* = 374), those in high LD (*n* = 234), and those in Hardy–Weinberg disequilibrium (*n* = 7), there were 158 remaining SNPs of interest distributed across the 43 genes (Supplementary Tables 2, 3 and 4).

Among the top 10 associated SNPs, rs4848627 in *GLI2* was found to be associated with development of any KC (OR = 1.53; 95%CI = 1.06–2.13, *P* = 4.2 × 10^–4^) and with development of SCCs exclusively (OR = 2.12; 95%CI = 1.39–3.23, *P* = 3.0 × 10^–4^) (Table 1).The *GLI2* SNP rs4848627 was also in the top SNPs associated with exclusive development of BCCs (OR = 1.38; 95%CI = 1.04–1.83, *P* = 0.021). Still other *GLI2* variants, rs895485 in intron 2 and rs17050378 in intron 1 were present among the top 10 associated SNPs for SCCs-only (*P* = 0.014 and 0.021, respectively; Table 1).

**Table 1:** SNP association analysis: The top 10 SNPs associated with participants who developed KC, and BCCs or SCCs exclusively

	**Chr**	**SNP**	**Gene name**	**A1**	**A2**	***P***	**OR**	**L95**	**U95**	**Permuted** **P**
**KC**	2	rs4848627	*GLI2*	G	A	0.0003	1.53	1.06	2.13	0.0004
	9	rs10869721	*PRKACG*	G	A	0.0028	1.43	1.13	1.81	0.0044
	12	rs3217882	*CCND2*	G	A	0.0141	1.29	1.05	1.58	0.0115
	2	rs41268683	*LRP2*	A	G	0.0151	1.72	1.10	2.67	0.0157
	7	rs7799478	*SMURF1*	A	G	0.0195	1.28	1.04	1.58	0.0209
	2	rs17050378	*GLI2*	A	G	0.0202	1.51	1.06	2.13	0.0171
	7	rs59136417	*GLI3*	A	G	0.0275	1.26	1.02	1.54	0.0302
	7	rs36113796	*GLI3*	A	C	0.0279	1.40	1.04	1.88	0.0215
	4	rs4689304	*EVC*	A	G	0.0308	1.25	1.02	1.54	0.0347
	9	rs4745514	*PRKACG*	A	G	0.0324	1.28	1.02	1.61	0.0260
**BCC**	12	rs3217882	*CCND2*	*G*	A	0.004	1.43	1.12	1.82	0.004
	1	rs970318	*PRKACB*	*A*	G	0.009	0.72	0.56	0.92	0.009
	9	rs10869721	*PRKACG*	*G*	A	0.013	1.43	1.07	1.89	0.017
	7	rs6954873	*SHH*	*A*	G	0.018	1.57	1.56	1.59	0.023
	2	rs4848627	*GLI2*	*G*	A	0.022	1.38	1.04	1.83	0.021
	1	rs7547892	*PRKACB*	*G*	A	0.025	0.76	0.59	0.96	0.029
	3	rs55814436	*BOC*	*A*	G	0.040	0.73	0.54	0.98	0.041
	4	rs2279247	*EVC*	*G*	A	0.043	0.75	0.57	0.99	0.076
	7	rs36113796	*GLI3*	*A*	C	0.044	1.43	1.00	2.02	0.035
	4	rs4689304	*EVC*	*G*	A	0.055	0.78	0.61	1.00	0.053
**SCC**	2	rs4848627	*GLI2*	*G*	A	0.0004	2.12	1.39	3.23	0.0003
	18	rs2849380	*BCL2*	*A*	G	0.0053	1.85	1.19	2.87	0.0075
	7	rs35280470	*GLI3*	*A*	G	0.0073	2.29	1.23	4.25	0.0064
	2	rs895485	*GLI2*	*A*	G	0.0143	0.60	0.40	0.91	0.0139
	2	rs17050378	*GLI2*	*A*	G	0.0269	1.94	107	3.51	0.0208
	4	rs735172	*EVC*	*G*	A	0.0274	1.58	1.05	2.37	0.0233
	7	rs114615136	*GLI3*	*G*	A	0.0294	1.85	1.05	3.25	0.0414
	3	rs13094203	*BOC*	*A*	G	0.0318	1.68	1.04	2.72	0.0284
	5	rs10058728	*CSNK1A1*	*A*	T	0.0440	1.50	1.01	2.23	0.0714
	3	rs73235151	*BOC*	*A*	C	0.0496	0.58	0.34	1.01	0.0743

*Chr* Chromosome, *A1* effect Allele, *A2* Non-effect Allele, *P* *P* value, *L95* 95% confidence interval (lower level), *U95%* 95% confidence interval (upper level)

In addition, we found an association with rs3217882, located in an intron of cyclin D2 (*CCND2*), which was associated with an increased risk of exclusive BCC development (OR 1.43; 95%CI 1.12–1.82, *P* = 0.004) and SNP rs970318 in *PRKACB* associated with exclusive BCC diagnosis (OR 0.72; 95% CI 0.56–0.92, *P* = 0.009).

SNP rs2849380 located in *BCL2* was the second most strongly associated SNP among exclusive SCC cases (OR = 1.85; 95%CI 1.19–2.87, *P* = 0.0075). Sensitivity analysis in which we excluded the 56 participants who potentially could have been misclassified as controls, showed very similar results (data not shown).

### Gene-based analysis

None of the top three genes associated with development of any KC, or exclusive development of either BCCs or SCCs respectively, reached statistical significance in our analysis (Table 2). *PRKACG* (protein kinase cAMP-activated catalytic subunit gamma) showed the strongest association among KC cases (*P* = 0.013; Z-score = 2.2) and was also the third strongest association in participants with exclusive BCC development (*P* = 0.012; Z-score = 1.7). The Sonic Hedgehog signaling molecule (*SHH*) was the top gene associated with BCCs-only cases (*P* = 0.005; Z-score 2.5). Kinesin family member 7 (*KIF7*) was the gene most strongly associated with SCCs-only cases (*P* = 0.008; Z-score 0.008).

**Table 2:** Gene association analysis. Top three genes associated with KC and exclusive development of BCCs or SCCs

KC vs Controls (total *n* = 795)					
**Chromosome**	**Gene**		**No of SNPs analysed in gene**	**Z score**	***P* value**
9	*PRKACG*	protein kinase cAMP-activated catalytic subunit gamma	4	2.2271	0.01297
3	*GSK3B*	glycogen synthase kinase 3 beta	29	1.5958	0.05526
7	*SHH*	sonic hedgehog signaling molecule	5	1.3246	0.09266
**BCCs exclusively vs Controls (total *n* = 670)**					
**Chromosome**	**Gene**		**No of SNPs analysed in gene**	**Z score**	***P* value**
7	*SHH*	sonic hedgehog signaling molecule	5	2.5477	0.00542
3	*GSK3B*	glycogen synthase kinase 3 beta	29	1.8958	0.02899
9	*PRKACG*	protein kinase cAMP-activated catalytic subunit gamma	4	1.7328	0.01214
**SCCs exclusively vs Controls (total *n* = 539)**					
**Chromosome**	**Gene**		**No of SNPs analysed in gene**	**Z score**	***P* value**
15	*KIF7*	kinesin family member 7	4	2.3964	0.00828
18	*BCL2*	BCL2 apoptosis regulator	73	2.2388	0.01258
5	*CSNK1G3*	casein kinase 1 gamma 3	8	2.0339	0.02098

## Discussion

We have previously shown that people living in Queensland with high UV exposure who develop multiple skin cancers over time are more likely to develop the same type of KC exclusively (BCCs-only or SCCs-only), than a mix of BCCs and SCCs tumors [21], suggesting a genetic component in the aetiology of much KC development. While many skin cancer genes are associated with both BCCs and SCCs, there are genes which are involved with either BCC or SCC development exclusively [27], yet to our knowledge, there are no prior human studies which have evaluated if genes involved in the SHH pathway affect the risk of development of BCCs alone or SCCs alone. In our study, *GLI2* was the gene that contained the most SNPs statistically significantly associated with any KC, as well as with exclusive development of BCCs or SCCs. The SNP with the strongest association with KC was rs4848627 in *GLI2* (1.5 times increased risk for any KC), and it was the top SNP for people who developed SCCs exclusively with a doubling of risk. This SNP was also found in the top 10 list of SNPs associated with exclusive development of BCCs.

*GLI2* plays an important role in regeneration and survival of keratinocytes, and the expression of *GLI2* together with *GLI1* are part of a positive feedback mechanism in BCC that leads to an increase in DNA-synthesis in confluent human keratinocytes [28]. Overexpression of *GLI2* supports epidermal regeneration in organotypic cell cultures, which over time lead to manifestation of undifferentiated basal cell-associated keratinocytes [29]. It has also been shown that *GLI* transcription factors can facilitate keratinocyte survival and transformation upon exposure to genotoxic agents. Because *GLI1* and *GLI2* facilitate the propagation of cells with damaged DNA, their expression may be naturally higher in cells that form the earliest precursor tumor lesions [30]. Transgenic mice overexpressing *GLI2* develop multiple BCCs and *GLI2* is required to maintain tumor growth [31]. While most of this evidence suggests a role of *GLI1* and *GLI2* expression in maintenance of keratinocyte integrity and avoidance of BCC formation, our genetic analyses particularly suggested associations with SCCs-only development which has not been explored before, as well as their relevance to BCC.

We further found that SNP rs3217882 in cyclin D2 (*CCND2)* was associated with a 1.4 times increased risk of exclusive BCCs. *CCND2* forms a complex which regulates the activity of cell cycle G1/S transition [32], which may partly explain why we found this SNP to be also associated with the overall KC group (1.3 times increased risk). There are no previous publications describing the association of KCs with this SNP.

Protein kinase cAMP-activated catalytic subunit gamma (*PRKACG*) was the gene with the second strongest association with KC overall, and it was also found in the top three gene list among people with exclusive. This gene has been linked to the bleeding disorder platelet-type 19 (BDPLT19) caused by platelet dysfunction [33]. Again there are no previous publications on skin cancer and *PRKACG* association, and repeated analyses in a larger cohort would be needed to confirm this possible association between the *PRKACG* gene and skin cancer.

The sonic hedgehog signaling molecule (*SHH* gene) was found to be associated with exclusive development of BCC. Defects in the SHH gene are otherwise known to cause a disorder characterised by facial deformities and problems with forebrain development in embryos [34]. Meanwhile, we found that kinesin family member 7 (*KIF7*) was the gene with the strongest association for exclusive development of SCCs. *KIF7* is an important gene in the SHH pathway, and acts as a negative regulator by preventing activation of *GLI2* in the absence of a ligand, and as a positive regulator by preventing the processing of *GLI3* into its repressor form. It is also involved in the regulation of epidermal differentiation [35] which is altered in skin cancer development [36].

A main strength of these genetic analyses was that they were based within the Nambour Skin Cancer Study population who have excellent phenotypic characterisation through prospective long-term (1992–2007) follow-up and histological confirmation of all skin cancers, which is not the case for most previous such studies where skin cancer phenotypes have not been routinely confirmed by histopathology. Thus we are uniquely positioned to analyse genetic associations in relation to the characterisation of participants’ long-term propensity to develop either BCCs or SCCs exclusively, as well as any KC, and this is the first study to do this for genes in the SHH pathway. Our study cohort consisted of an ethnically homogenous population (mostly European ancestry), which minimises the chance of false positive associations due to population substructure. The major limitation of this study was the relatively small sample size resulting in a lack of statistical power, and hence we have focussed on the interpretation of top SNP and gene hits only, and have explored potential underlying mechanisms relevant to BCC and SCC development.

## Conclusion and implications

Our study focussed on SNP and gene-associations of the SHH pathway in people who develop KC, including a novel long-term characterisation of propensity to develop BCCs or SCCs exclusively. We found *GLI2* to be significantly associated with exclusive SCC development as well as development of KC overall. Further, we identified associations of other SNPs and genes in the SHH pathway, warranting larger longitudinal studies to replicate and build on these findings.

## Supplementary Information

**Additional file 1:** **Supplementary Table 1.** General characteristics of controls and cases (total, BCC only, SCC only, and BCC and SCC).** Supplementary Table 2.** Association of gene variants in the SHH pathway with KC. We tested the association of 158 SNPs distributed across 43 genes involved in the SHH pathway with KC (BCC, SCC, BCC, and SCC). SNPs are presented in ascending order by p-value. Information on the chromosome location (CHR), SNP name (SNP), base pair position along the chromosome (BP), the allele with the minor allele frequency (A1), allele 2 (A2), A1 frequency in cases (F_cases), and controls (F_controls), Chi-square value (CHISQ), p-value (P) and odds ratio (OR) are presented. **Supplementary Table 3.** Association of gene variants in the SHH pathway with BCC. We tested the association of 158 SNPs distributed across 43 genes involved in the SHH pathway with BCC.  SNPs are presented in ascending order by p-value. Information on the chromosome location (CHR), SNP name (SNP), base pair position along the chromosome (BP), the allele with the minor allele frequency (A1), allele 2 (A2), A1 frequency in cases (F_cases) and controls (F_controls), Chi-square value (CHISQ), p-value (P) and odds ratio (OR) are presented. **Supplementary Table 4. **Association of gene variants in the SHH pathway with SCC. We tested the association of 158 SNPs distributed across 43 genes involved in the SHH pathway with SCC.  SNPs are presented in ascending order by p-value. Information on the chromosome location (CHR), SNP name (SNP), base pair position along the chromosome (BP), the allele with the minor allele frequency (A1), allele 2 (A2), A1 frequency in cases (F_cases) and controls (F_controls), Chi-square value (CHISQ), p-value (P) and odds ratio (OR) are presented. **Supplementary Table 5.** Sonic Hedgehog pathway genes.

## Data Availability

All data generated or analysed during this study are available at the Queensland University of Technology (QUT) repository, https://doi.org/10.25912/RDF_1646199819849.

## Abbreviations

BCC: Basal cell carcinoma
HWE: Hardy Weinberg Equilibrium
KC: Keratinocyte cancer
MAF: Minor allele frequency
LD: Linkage disequilibrium
QC: Quality control
SCC: Cutaneous squamous cell carcinoma
SHH: Sonic Hedgehog
SNP: Single nucleotide polymorphism
UV: Ultraviolet radiation
