# Supplementary material for: Associations of keratinocyte cancers with snp variants in the sonic hedgehog pathway
Source: BMC Cancer. 2022 May 3;22:490. doi: 10.1186/s12885-022-09565-6 (PMC9063108; doi:10.1186/s12885-022-09565-6)
Supplement: Supplementary file 1 — Additional file 1: Supplementary Table 1. General characteristics of controls and cases (total, BCC only, SCC only, and BCC and SCC). Supplementary Table 2. Association of gene variants in the SHH pathway with KC. We tested the association of 158 SNPs distributed across 43 genes involved in the SHH pathway with KC (BCC, SCC, BCC, and SCC). SNPs are presented in ascending order by p-value. Information on the chromosome location (CHR), SNP name (SNP), base pair position along the chromosome (BP), the allele with the minor allele frequency (A1), allele 2 (A2), A1 frequency in cases (F_cases), and controls (F_controls), Chi-square value (CHISQ), p-value (P) and odds ratio (OR) are presented. Supplementary Table 3. Association of gene variants in the SHH pathway with BCC. We tested the association of 158 SNPs distributed across 43 genes involved in the SHH pathway with BCC. SNPs are presented in ascending order by p-value. Information on the chromosome location (CHR), SNP name (SNP), base pair position along the chromosome (BP), the allele with the minor allele frequency (A1), allele 2 (A2), A1 frequency in cases (F_cases) and controls (F_controls), Chi-square value (CHISQ), p-value (P) and odds ratio (OR) are presented. Supplementary Table 4. Association of gene variants in the SHH pathway with SCC. We tested the association of 158 SNPs distributed across 43 genes involved in the SHH pathway with SCC. SNPs are presented in ascending order by p-value. Information on the chromosome location (CHR), SNP name (SNP), base pair position along the chromosome (BP), the allele with the minor allele frequency (A1), allele 2 (A2), A1 frequency in cases (F_cases) and controls (F_controls), Chi-square value (CHISQ), p-value (P) and odds ratio (OR) are presented. Supplementary Table 5. Sonic Hedgehog pathway genes. [file 12885_2022_9565_MOESM1_ESM.zip › SHH- supplementary_material_14Dec.docx]

**ASSOCIATIONS OF KERATINOCYTE CANCERS WITH SNP VARIANTS IN THE SONIC HEDGEHOG PATHWAY**

**Authors:**

Astrid J. Rodriguez-Acevedo^1^*, Annika Antonsson^1,2^*, Upekha E. Liyanage^1^, Maria Celia B. Hughes^1^, Scott Gordon^1^, , Jolieke C. van der Pols^3^ and Adele C. Green^1,4^

*equal contribution

^1^ QIMR Berghofer Medical Research Institute, Brisbane, Australia

^2^ Faculty of Medicine, The University of Queensland, Brisbane, Australia

^3^ Queensland University of Technology (QUT), Faculty of Health, School of Exercise and Nutrition Sciences, Brisbane, Australia

^4^ CRUK Manchester Institute and Faculty of Biology Medicine and Health, University of Manchester, Manchester Academic Health Science Centre, Manchester, UK

**Supplementary Table 1: General characteristics of controls and cases (total, BCC only, SCC only, and BCC and SCC).**

|  | **Category** | **Controls (N=484)**  **n (%)** | **Total Cases (N=311)**  **n (%)** | **BCC only (N=186)**  **n (%)** | **SCC only**  **(N=55)**  **n (%)** | **BCC & SCC (N=70)**  **n (%)** |
| --- | --- | --- | --- | --- | --- | --- |
| Age, years | 20-39 | 151 (31.2) | 37 (11.8)* | 29 (15.6) | 5 ( 9.1) | 3 ( 4.3) |
|  | 40-49 | 155 (32.0) | 74 (23.7)* | 57 (30.6) | 7 (12.7) | 10 (14.3) |
|  | 50-59 | 98 (20.2) | 74 (23.7) | 47 (25.3) | 9 (16.4) | 18 (25.7) |
|  | 60-70 | 80 (16.5) | 126 (40.5)* | 53 (28.5) | 34 (61.8) | 39 (55.7) |
| Sex | Female | 287 (59.3) | 164 (52.7) | 105 (56.5) | 29 (52.7) | 30 (42.9) |
|  | Male | 197 (40.7) | 147 (47.3) | 81 (43.5) | 26 (47.3) | 40 (57.1) |
| Skin colour (determined by dermatologist) | Fair | 193 (40.0) | 162 (52.1)* | 85 (46.2) | 35 (63.6) | 42 (60.0) |
|  | Medium | 226 (46.9) | 128 (41.1) | 86 (46.7) | 18 (32.7) | 24 (34.3) |
|  | Olive | 61 (12.7) | 19 (0.06) * | 13 ( 7.1) | 2 ( 3.6) | 4 ( 5.7) |
|  | Black | 2 ( 0.4) | 0 (0) | 0 ( 0.0) | 0 ( 0.0) | 0 ( 0.0) |
| skin reaction to acute sun (Self-reported) | Always burn | 71 (14.7) | 92 (29.6)* | 43 (23.1) | 25 (45.5) | 24 (34.3) |
|  | Burn then tan/only tan | 412 (85.3) | 219 (70,4)* | 143 (76.9) | 30 (54.5) | 46 (65.7) |
| Leisure activities | Mainly outdoors | 200 (41.5) | 148 (47.5) | 85 (45.7) | 27 (49.1) | 36 (51.4) |
|  | Indoors/outdoors | 215 (44.6) | 119 (38.3) | 71 (38.2) | 20 (36.4) | 28 (40.0) |
|  | Mainly indoors | 67 (13.9) | 44 (14.1) | 30 (16.1) | 8 (14.5) | 6 ( 8.6) |
| Lifetime painful sunburns | Never | 52 (10.8) | 28 (0.9) | 18 ( 9.7) | 3 ( 5.5) | 7 (10.0) |
|  | Once | 101 (20.9) | 57 (18.3) | 30 (16.1) | 13 (23.6) | 14 (20.0) |
|  | 2-5 | 206 (42.7) | 131 (0.42) | 84 (45.2) | 18 (32.7) | 29 (41.4) |
|  | 5+ | 124 (25.7) | 95 (30.5) | 54 (29.0) | 21 (38.2) | 20 (28.6) |
| Clinical elastosis of neck | None | 133 (27.5) | 32 (0.10)* | 27 (14.6) | 4 ( 7.4) | 1 ( 1.4) |
|  | Low to moderate | 223 (46.1) | 150 (48.2) | 100 (54.1) | 24 (44.4) | 26 (37.7) |
|  | Severe | 128 (26.4) | 126 (40.5)* | 58 (31.4) | 26 (48.1) | 42 (60.9) |
| Sunscreen trial allocation | Discretionary sunscreen | 254 (52.5) | 160 (51.4) | 89 (47.8) | 30 (54.5) | 41 (58.6) |
|  | Daily sunscreen | 230 (47.5) | 151 (48.5) | 97 (52.2) | 25 (45.5) | 29 (41.4) |
| Beta-carotene trial allocation | Placebo | 233 (48.1) | 147 (47.2) | 89 (47.8) | 25 (45.5) | 33 (47.1) |
|  | Daily Beta-carotene | 251 (51.9) | 164 (52.7) | 97 (52.2) | 30 (54.5) | 37 (52.9) |

***** P-value < 0.05 for a Chi-square test for equality of proportions between cases and controls.

**Supplementary Table 2: Association of gene variants in the SHH pathway with KC.** We tested the association of 158 SNPs distributed across 43 genes involved in the SHH pathway with KC (BCC, SCC, BCC, and SCC). SNPs are presented in ascending order by p-value. Information on the chromosome location (CHR), SNP name (SNP), base pair position along the chromosome (BP), the allele with the minor allele frequency (A1), allele 2 (A2), A1 frequency in cases (F_cases), and controls (F_controls), Chi-square value (CHISQ), p-value (P) and odds ratio (OR) are presented.

Provided as an excel file

**Supplementary Table 3: Association of gene variants in the SHH pathway with BCC.** We tested the association of 158 SNPs distributed across 43 genes involved in the SHH pathway with BCC. SNPs are presented in ascending order by p-value. Information on the chromosome location (CHR), SNP name (SNP), base pair position along the chromosome (BP), the allele with the minor allele frequency (A1), allele 2 (A2), A1 frequency in cases (F_cases) and controls (F_controls), Chi-square value (CHISQ), p-value (P) and odds ratio (OR) are presented.

Provided as an excel file

**Supplementary Table 4: Association of gene variants in the SHH pathway with SCC.** We tested the association of 158 SNPs distributed across 43 genes involved in the SHH pathway with SCC. SNPs are presented in ascending order by p-value. Information on the chromosome location (CHR), SNP name (SNP), base pair position along the chromosome (BP), the allele with the minor allele frequency (A1), allele 2 (A2), A1 frequency in cases (F_cases) and controls (F_controls), Chi-square value (CHISQ), p-value (P) and odds ratio (OR) are presented.

Provided as an excel file

**Supplementary Table 5: Sonic Hedgehog pathway genes**

Provided as an excel file
